# Venetoclax combined with azacitidine in the treatment of secondary myelodysplastic syndrome following multiple myeloma: a case report and literature review

**DOI:** 10.3389/fonc.2026.1823066

**Published:** 2026-07-14

**Authors:** Lijun Shi, Zhongrui Ma, Xia Yu, Tian Wang, Li Wei, Yaning Pan, Tantian Jiang, Xiujin Wu

**Affiliations:** 1 Department of Hematology, Chengdu Fifth People’s Hospital, Chengdu, China; 2 Department of of Psychosomatic Medicine, Chengdu Fifth People’s Hospital, Chengdu, China

**Keywords:** azacitidine, multiple myeloma, myelodysplastic syndrome, treatment-related, venetoclax

## Abstract

We conducted a retrospective analysis of the clinical data of a patient with myelodysplastic syndrome (MDS) secondary to stable multiple myeloma (MM), who was treated with a combination of venetoclax and azacitidine at the Fifth People’s Hospital of Chengdu. Additionally, we reviewed the relevant literature. The patient, a 74-year-old male, was initially diagnosed with multiple myeloma (IgA-γ, DS IIIA, ISS III, R-ISS II) and achieved a very good partial response (VGPR) following sequential treatments with BCTD, RVD, and PVD regimens, subsequently receiving maintenance therapy with pomalidomide. Despite being in continuous remission, the patient developed secondary myelodysplastic syndrome characterized by refractory anemia with excess blasts-2 (MDS-RAEB-II) 51 months post-initial MM diagnosis. He was then treated with venetoclax in combination with azacitidine. After the first treatment course, the patient achieved morphological partial remission of MDS. However, due to non-adherence to continuous treatment, he ultimately succumbed to a secondary infection.

## 1 Introduction

Multiple myeloma (MM) is a hematological malignancy characterized by the neoplastic proliferation of monoclonal plasma cells within the bone marrow, accompanied by the secretion of monoclonal immunoglobulins or their fragments (M protein), which leads to subsequent damage to associated organs or tissues. In recent years, the widespread implementation of novel therapeutic approaches, including immunomodulatory agents, proteasome inhibitors, and monoclonal antibodies, has markedly enhanced the survival rates of patients with MM (1). Nonetheless, long-term survivors of MM are susceptible to treatment-related chronic complications, notably therapy-related myelodysplastic syndrome (t-MDS) and therapy-related acute myeloid leukemia (t-AML), which occur at significantly higher incidence rates compared to the general population. This has emerged as a critical concern impacting the long-term survival of MM patients (2). t-MDS/AML typically manifest following chemotherapy or radiotherapy and are associated with greater malignancy and poorer prognosis compared to primary MDS/AML (3, 4). Currently, the treatment of therapy-related myelodysplastic syndromes (t-MDS) primarily relies on patient risk stratification and includes approaches such as hematopoietic stem cell transplantation, the use of demethylating agents like azacitidine, and chemotherapy regimens (5). The combination of venetoclax (VEN) with azacitidine has demonstrated efficacy in primary myelodysplastic syndromes (MDS) and acute myeloid leukemia (AML); however, its use in patients with secondary t-MDS following multiple myeloma (MM) remission has been infrequently documented both domestically and internationally. This article retrospectively examines the clinical data of a patient with secondary t-MDS post-MM remission, who was admitted to the Department of Hematology at the Fifth People’s Hospital of Chengdu in March 2020 and treated with the VEN and azacitidine regimen. Additionally, it reviews relevant literature to provide a reference for the clinical management of similar cases.

## 2 Case report

On March 17, 2020, a 74-year-old male patient was admitted to the hematology department of our hospital, presenting with a one-month history of chest and back pain accompanied by fatigue. The patient’s medical, personal, and family histories were unremarkable. Upon admission, the examination revealed a body temperature of 36.8 °C, a heart rate of 78 beats per minute, a respiratory rate of 21 breaths per minute, and blood pressure of 116/68 mmHg (1 mmHg = 0.133 kPa). The patient presented with pallor suggestive of anemia, and no superficial lymphadenopathy was palpable upon physical examination. Bilateral pulmonary auscultation revealed coarse respiratory sounds, in the absence of dry or moist rales. Abdominal and cardiovascular assessments were unremarkable, and no edema was observed in the lower limbs.

Laboratory studies demonstrated a hemoglobin level of 84 g/L, a white blood cell count of 6.85 ×10^9^/L, with neutrophils measuring 2.8×10^9^/L, and a platelet count of 417×10^9^/L. Biochemical analysis indicated a globulin concentration of 60.5 g/L and an albumin level of 31 g/L. Additional parameters included a urea level of 9.0 mmol/L, serum creatinine of 70.4μmol/L, serum calcium of 2.16 mmol/L, β2-microglobulin of 5.2 mg/L, and lactate dehydrogenase of 165 U/L. Chest and abdominal X-rays indicated changes in the bilateral ribs, bilateral iliac bones, and the pubic and ischial bones.The spinal MRI reveals degenerative changes in the cervical, thoracic, and lumbar vertebrae, characterized by extensive bone resorption and multiple vertebrae exhibiting varying degrees of flattening, with some showing recent compression changes. Serum protein electrophoresis indicates the presence of an M protein band, with an M protein concentration of 13.12%. Abnormal plasma cells constitute 68% of the nucleated cells. Flow cytometry immunotyping demonstrates partial expression of CD38 and CD138, with no expression of CD19 and CD56. There is a restricted expression of intracellular immunoglobulin Lambda light chain, and approximately 79.1% of the plasma cells are monoclonal. The TP53 gene is negative. Fluorescence *in situ* hybridization (FISH) results are negative for 1q21, IG/MAF, IG/MAFB, IG/FGFR3, and P53/CEP17. Chromosomal karyotyping shows a normal male karyotype of 46, XY [20]. Both chest CT and cardiac color Doppler ultrasound reveal no significant abnormalities.The patient was diagnosed with multiple myeloma, specifically of the IgA-γ light chain type, classified as Durie-Salmon stage IIIA, International Staging System (ISS) stage III, and Revised ISS (R-ISS) stage II. The patient also presented with compression fractures in the thoracic and lumbar vertebrae and osteoporosis. Initially, symptomatic supportive treatment was provided, followed by chemotherapy using the BCD regimen (bortezomib 2 mg on days 1, 8, 15, and 22; cyclophosphamide 0.4 g on days 1, 8, 15, and 22; dexamethasone 15 mg on days 1-2, 8-9, 15-16, and 22-23) from April to July 2020. After four cycles, the patient’s condition was assessed as a partial response (PR). In August 2020, treatment was transitioned to the RVD regimen (lenalidomide 25 mg on days 1-21; bortezomib 2 mg on days 1, 8, 15, and 22; dexamethasone 10 mg on days 1-2, 8-9, 15-16, and 22-23). Following four cycles of the RVD regimen, the patient achieved a very good partial response (VGPR). The RVD regimen was continued, and the patient’s condition was repeatedly evaluated as stable. In February 2023, an evaluation of the patient’s condition indicated a symptomatic recurrence of the disease. A treatment regimen incorporating carfilzomib or daratumumab was recommended. However, due to financial constraints and a preference for oral medication, the patient and their family consented only to transition to the PVD regimen, consisting of pomalidomide (25 mg, administered on days 1-21), bortezomib (2 mg, administered on days 1, 8, 15, and 22), and dexamethasone (10 mg, administered on days 1-2, 8-9, 15-16, and 22-23) for chemotherapy. Subsequent evaluations demonstrated that the patient achieved a Very Good Partial Response (VGPR).

**Figure 1:**
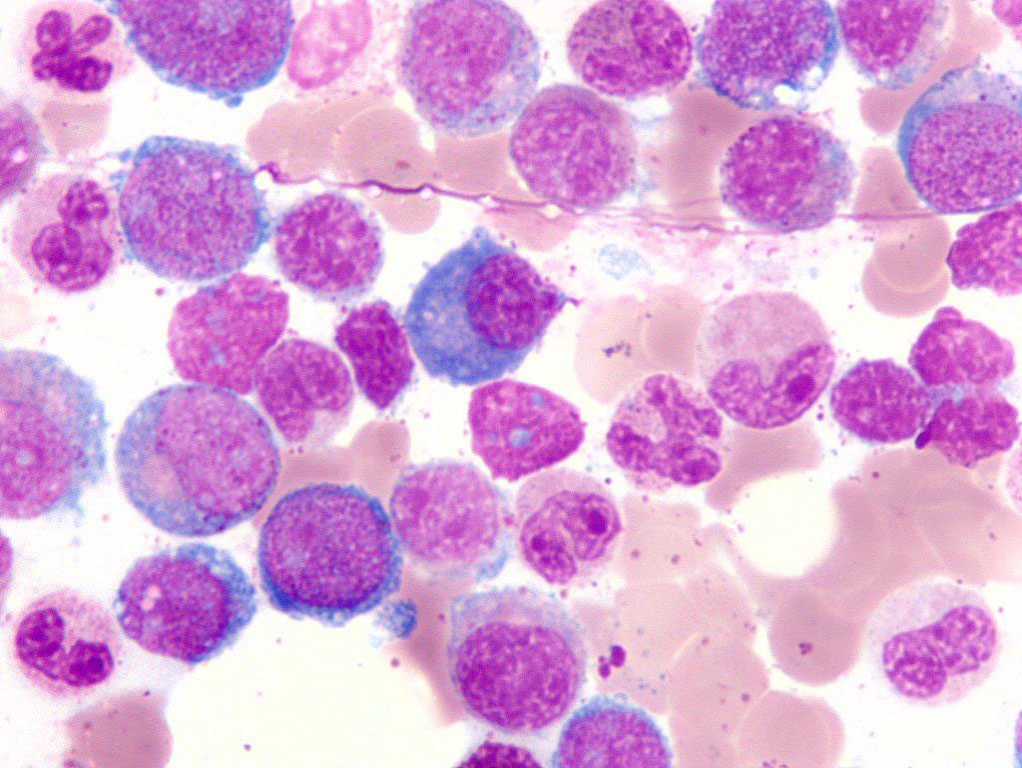
Bone marrow smear examination results of patients with myelodysplastic syndrome complicated with multiple myeloma. Arrow indicates blast cells. (Wright ×1000).

In June 2024, the patient was admitted to the hospital for a comprehensive medical evaluation. The complete blood count revealed the following: hemoglobin level at 58 g/L, white blood cell count at 2.85 × 10^9^/L, neutrophil count at 2.35 × 10^9^/L, and platelet count at 7 × 10^9^/L. Morphological analysis of bone marrow cells indicated that nucleated cell proliferation was markedly active. The granulocyte series exhibited abnormal proliferation, constituting 89% of nucleated cells, within which blasts comprised 16%,cells exhibiting nuclear-cytoplasmic asynchrony were identified. The plasma cell proportion was within the normal range, with occasional immature forms observed. Erythroid proliferation was reduced, with no significant morphological abnormalities noted. The lymphocyte proportion was decreased, with generally normal morphology. Approximately 11 megakaryocytes were identified in the entire smear, accompanied by scattered thrombocytopenia (**Figure 1**). Flow cytometry immunophenotyping identified abnormal primitive/immature myeloid cells comprising approximately 18.18%, expressing CD34, CD117, HLA-DR, partial CD13, and partial CD33, while lacking expression of MPO, CD38, CD138, CD56, and CD19. Monoclonal plasma cells accounted for approximately 3.85%, expressing CD38 and CD138, but not expressing CD19 or CD56, with restricted expression of intracellular immunoglobulin Lambda light chain. Genetic analysis revealed mutations in common myeloid genes, including SETBP1 gene p.D868N, ASXL1 p.G646fs, RUNX1 gene p.F116fs, ZRSR2 gene p.R126, and KRAS gene p.A59G. No common fusion genes were detected. Serum protein electrophoresis demonstrated an M protein level of 1.36% within a total protein concentration of 48.6 g/L.The patient’s diagnosis includes: 1. Treatment-related myelodysplastic syndrome with refractory anemia and excess blasts (MDS-RAEB-II) with an IPSS score of 2, IPSS-R score of 6.5, and IPSS-M score of 3.8; 2. Multiple myeloma(IgA-γ type, DS stage-IIIA, ISS stage-III,R-ISS stage-II); 3. Compression fractures of the thoracic and lumbar vertebrae.

**Figure 2:**
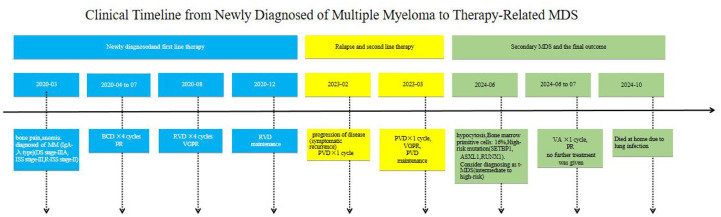
Clinical timeline of multiple myeloma (MM) and subsequent therapy-related myelodysplastic syndrome (t-MDS).

The patient had a history of multiple myeloma for 51 months. Currently, based on bone marrow cytology and flow cytometry, the diagnosis was MDS, which was considered as t-MDS. The prognosis stratification was intermediate to high-risk. Due to the patient’s old age and poor tolerance, intensified chemotherapy was not suitable. Therefore, the VA regimen (V: 100mg on days 1-21, A: 100mg on days 1-7) was administered for 28 days. Supportive treatments such as blood product transfusion and anti-infection were also given. During the treatment, the patient experienced 3-4 grade hematological adverse reactions for 21 days. Two weeks after the end of the treatment, the bone marrow morphology examination showed that the proportion of promyelocytes was high, accounting for 5.5% of ANC. The results of bone marrow flow cytometry: abnormal primitive/immature myeloid cells accounted for 4.47% of nucleated cells. The re-examination of the blood routine test showed that the hemoglobin level was 76 g/L, the white blood cell count was 1.26×10^9^/L, the neutrophil count was 0.97×10^9^/L, and the platelet count was 64×10^9^/L.Blood and urine electrophoresis: M protein 1.26% (total protein 49.6g/L).The assessment of MDS treatment response indicated partial response (PR). It was recommended that the patient continue with demethylation treatment, but the patient refused. Outpatient oral Chinese medicine treatment was given. During the follow-up period, the family was notified that the patient had succumbed to a severe pulmonary infection in October 2024 (**Figure 2**).

## Discussion

With the continuous progress and wide application of new drugs in the treatment of multiple myeloma (MM), the survival period of patients has significantly increased. However, long-term disease control and continuous anti-myeloma treatment also bring an increased risk of secondary hematological malignancies. Among them, treatment-related myelodysplastic syndrome (t-MDS) is one of the most challenging adverse events in clinical practice (6). Compared with primary MDS, t-MDS has more aggressive biological characteristics, impaired hematopoietic reserves, complex molecular and cytogenetic abnormalities, and a poorer overall prognosis (3, 4, 7, 8). This patient developed myelodysplastic syndrome with increased blasts - type II (MDS-RAEB-II) 51 months after being diagnosed with IgA-type MM and receiving multiple lines of treatment. This course of disease is consistent with the high incidence period of t-MDS and treatment-related acute myeloid leukemia (t-AML) as reported in the literature (3.5-5.0 years after initiation of anti-myeloma therapy) (9). Considering the patient’s long history of multi-drug treatment for MM, refractory pancytopenia, and increased proportion of bone marrow blasts, the final diagnosis was MM-related t-MDS. This case not only confirmed the epidemiological association between long-term treatment of MM and secondary myeloid tumors, but also because it presents several unique characteristics different from previous reported cases, thus providing new clinical implications and academic value for this case report.

The specific mechanism of MM leading to MDS has not been fully elucidated yet. It may be the result of multiple factors acting together (9). Firstly, the cumulative effect of chemotherapy drugs is an important risk factor. This patient received a multi-drug combination regimen containing alkylating agents, immunomodulators, and proteasome inhibitors during MM treatment. Agents such as cyclophosphamide, an alkylating agent, can directly bind to DNA, causing continuous gene mutations and chromosomal instability (10–14); lenalidomide may increase the mutation risk of normal hematopoietic stem cells through the CRBN pathway (15); while bortezomib inhibits autophagy and disrupts the metabolic homeostasis of stem cells (16). The long-term cumulative damage of these drugs gradually impairs the normal hematopoietic function of the bone marrow. Secondly, the inflammatory microenvironment may play an important role in MM-induced MDS. IL-6 (17), as a key inflammatory factor, can antagonize drug-induced tumor cell apoptosis in MM, but disrupts the proliferation and differentiation of hematopoietic stem cells and progenitor cells in secondary MDS, ultimately creating a favorable microenvironment for the expansion of malignant mutation clones. In this case, the cumulative DNA damage and the chronic IL-6-driven inflammation may promote the clonal selection and proliferation of hematopoietic stem cells carrying SETBP1, ASXL1, RUNX1, and other adverse mutations, ultimately leading to the occurrence of high-risk t-MDS. Moreover, repeated immune responses may drive the transformation of stem cell clones (12, 13). Previous studies have shown (10, 15, 18–21) that the presence of clonal hematopoiesis such as TP53 mutations and del(20q) or del(5q) as clonal events is the most common driving events and adverse cytogenetic abnormalities in t-MDS. However, in this patient, no TP53 mutations or chromosomal abnormalities were found at the time of MM diagnosis, and no TP53 mutations were detected through limited genetic testing after the occurrence of secondary t-MDS. Nevertheless, five somatic mutations in myeloid-related genes were identified through genetic testing, including SETBP1 p.D868N, ASXL1 p.G646fs, RUNX1 p.F116fs, ZRSR2 p.R126, and KRAS p.A59G. These mutations involve multiple functional pathways, including epigenetic regulation, RNA splicing, hematopoietic transcriptional regulation, and oncogenic signal transduction, which constitute the typical molecular features of high-risk t-MDS. These findings are the main innovation points of this case. Our observations confirm that in the absence of TP53 mutations and other adverse cytogenetic abnormalities, the gradual accumulation of multiple high-risk myeloid driver mutations may promote the progression of MM to high-risk t-MDS, which further highlights the significant genetic heterogeneity of this disease. From a clinical perspective, for long-term surviving MM patients, even if the basic TP53 and karyotype are normal, regular dynamic monitoring of myeloid molecular mutations is crucial for early identification and intervention of secondary t-MDS. However, this study has limitations in cytogenetic assessment. After diagnosing t-MDS, routine chromosome karyotype analysis and FISH detection for del(20q) and others were not repeated, so potential latent chromosomal abnormalities could not be completely excluded.

Currently, there is no unified standard for the treatment of MM secondary to t-MDS. Due to the previous treatment history, immunosuppressive state, and possible macrophage dysfunction in t-MDS patients, they have poor tolerance to conventional chemotherapy and are prone to severe infections, resulting in extremely poor prognosis (6). Demethylating drugs such as azacitidine have become the first-line treatment for intermediate-high risk t-MDS (22, 23), but the efficacy of single-agent treatment is limited in patients with complex molecular abnormalities. In recent years, the VA regimen (venetoclax combined with azacitidine) has achieved encouraging efficacy in MDS and AML (24–28), but there are few reports of its use in MM secondary t-MDS. In this case, we attempted to treat the patient with venetoclax combined with azacitidine. After one course of treatment, the percentage of bone marrow blasts decreased from 16% to 5.5%, the proportion of abnormal myeloid precursor cells detected by flow cytometry decreased from 18.18% to 4.47%, and peripheral blood cell reduction was significantly improved, achieving partial remission in morphology and hematology. The rapid and transient disease response of this patient can be attributed to the complementary and collaborative effect of this regimen (29) and the possibility that venetoclax may to some extent overcome the HMA resistance caused by adverse molecular lesions (30). This suggests that the VA regimen can be an effective treatment option for elderly, frail patients with high-risk t-MDS who have mutations. Although hematological adverse reactions and pulmonary fungal infections occurred during the treatment, the adverse events were well controlled after supportive treatment including blood transfusion and standardized anti-infection therapy, indicating that the safety of this regimen is controllable under strict clinical monitoring and comprehensive supportive care. However, this study is only a single-case observation and cannot draw definite conclusions on the long-term remission rate, overall survival benefit, and general safety of the VA regimen in t-MDS. The patient eventually died of severe secondary pulmonary infection, mainly due to the discontinuation of standardized demethylation treatment and the switch to alternative traditional Chinese medicine treatment outside the hospital. This result indicates that poor treatment compliance is also an important factor contributing to the poor prognosis of t-MDS patients. In clinical practice, it is crucial to communicate fully with patients and their families, emphasizing the necessity of continuous standardized treatment.

In conclusion, as the survival period of multiple myeloma patients has extended, it is crucial for clinical practice to pay close attention to the risk of developing secondary tumors, including treatment-related myelodysplastic syndrome. For patients who experience unexplained blood cell reduction or recurrence during treatment, timely completion of bone marrow cell morphology, flow cytometry, cytogenetics, and molecular biology tests is necessary to achieve the early diagnosis of secondary myeloid neoplasia in multiple myeloma. In this case, the VA regimen demonstrated good short-term efficacy and acceptable safety, providing new clinical ideas for the treatment of secondary myeloid dysplasia in multiple myeloma. However, given the inherent limitations of single-case studies, the effectiveness and safety of this regimen still need to be further verified by larger sample size clinical studies. In the future, by deeply exploring its molecular mechanism, searching for potential predictive markers and therapeutic targets, it will help optimize clinical decisions and improve patient prognosis.

## Data Availability

The datasets presented in this study can be found in online repositories. The names of the repository/repositories and accession number(s) can be found in the article/Supplementary Material.
